# Bioinformatics driven discovery of small molecule compounds that modulate the FOXM1 and PPARA pathway activities in breast cancer

**DOI:** 10.1038/s41397-022-00297-1

**Published:** 2022-11-24

**Authors:** Shujun Huang, Pingzhao Hu, Ted M. Lakowski

**Affiliations:** 1 College of Pharmacy, University of Manitoba, Apotex Centre, 750 McDermot Avenue, Winnipeg, MB R3E 0T5 Canada; 2 Department of Biochemistry, Schulich School of Medicine & Dentistry, Western University, London, ON N6A 5C1 Canada; 3 Research Institute in Oncology and Hematology, CancerCare Manitoba, 675 McDermot Aveune, Winnipeg, MB R3E 0T5 Canada

**Keywords:** Gene regulatory networks, Predictive medicine, Cancer genomics

## Abstract

Our previous studies demonstrated that the FOXM1 pathway is upregulated and the PPARA pathway downregulated in breast cancer (BC), and especially in the triple negative breast cancer (TNBC) subtype. Targeting the two pathways may offer potential therapeutic strategies to treat BC, especially TNBC which has the fewest effective therapies available among all BC subtypes. In this study we identified small molecule compounds that could modulate the PPARA and FOXM1 pathways in BC using two methods. In the first method, data were initially curated from the Connectivity Map (CMAP) database, which provides the gene expression profiles of MCF7 cells treated with different compounds as well as paired controls. We then calculated the changes in the FOXM1 and PPARA pathway activities from the compound-induced gene expression profiles under each treatment to identify compounds that produced a decreased activity in the FOXM1 pathway or an increased activity in the PPARA pathway. In the second method, the CMAP database tool was used to identify compounds that could reverse the expression pattern of the two pathways in MCF7 cells. Compounds identified as repressing the FOXM1 pathway or activating the PPARA pathway by the two methods were compared. We identified 19 common compounds that could decrease the FOXM1 pathway activity scores and reverse the FOXM1 pathway expression pattern, and 13 common compounds that could increase the PPARA pathway activity scores and reverse the PPARA pathway expression pattern. It may be of interest to validate these compounds experimentally to further investigate their effects on TNBCs.

## Introduction

Breast cancer (BC) is the most common cancer and the second leading cause of death from cancer among Canadian women, accounting for 25% of all new cancer cases and 13% of cancer-related deaths [1]. As a heterogenous disease, BC can be divided into four major molecular subtypes: luminal A, luminal B, HER2-enriched, and triple negative breast cancer (TNBC) [2]. The TNBC subtype is chracterized by lacking expression of the estrogen receptor (ER), progesterone receptor (PR), and human epidermal growth factor receptor 2 (HER2), representing 5-10% of all breast cancers [3]. TNBCs show aggressive features such as high grade and high proliferation [4]. Patients diagnosed with TNBCs have a poor prognosis, and almost 40% of patients experience a relapse within 5 years post-diagnosis [4, 5]. Unlike breast tumors expressing ER and/or HER2, TNBCs generally do not respond to otherwise highly effective therapies such as selective estrogen receptor modulators (SERMs), like tamoxifen, raloxifene, and aromatase inhibitors that target the ER, or trastuzumab that targets HER2 expressing tumors [2]. Thus, finding effective therapeutic strategies for TNBCs is particularly challenging for researchers.

In one of our previous studies [6], the Gene Set Enrichment Analysis (GSEA) showed that the FOXM1 pathway was upregulated in all BC subtypes versus normal breast tissue samples and was the top upregulated pathway in TNBC. The GSEA results also demonstrated that the PPARA pathway was highly downregulated in all BC subtypes relative to normal and was the top downregulated pathway in TNBC samples. In another study [7], our integrative analyses revealed 25, 20, 15 and 24 key TF and miRNA regulators in luminal A, luminal B, HER2-enriched and TNBC subtypes, respectively. Two TFs and seven miRNAs were identified in all four subtypes and thus were referred to as common regulators. Gene set over-representation analysis of targets of the key regulators was performed to investigate pathways potentially regulated by these regulators. miR-340-5p and E2F1 were two common regulators found to be regulating PID_FOXM1_PATHWAY (also referred to as FOXM1 pathway in this study). miR-340-5p and another common regulator miR-664b-3p were found to be regulators of BIOCARTA_PPARA_PATHWAY (referred to as PPARA pathway in this study). Moreover, three other regulators (PPARA, PPARG, and miR-129-5p), which were identified in TNBC and together in one or two other subtypes, were found to regulate the PPARA pathway. miR-9-3p, which was identified as a key regulator in TNBC alone, was found to regulate the PPARA pathway.

The FOXM1 pathway is a predefined pathway extracted from the C2 category gene sets in the MSigDB [8]. The FOXM1 pathway is involved in cell cycle control and DNA damage repair, and it ultimately promotes tumor cell proliferation. A total of 40 gene members are engaged in this pathway, including tumor suppressors (e.g., *BRCA2*, *CDKN2A*, *CHEK2* and *RB1*), the proto-oncogene family *MYC*, genes encoding cyclins (e.g., *CCNA2*, *CCNB1*, *CCNB2*, *CCND1* and *CCNE1*), genes encoding cyclin-dependent kinases (e.g., *CDK1*, *CDK2* and *CDK4*), *ESR1*, *NEK2* as well as *FOXM1* itself. FOXM1 is one of the most important oncogenic TFs and it is overexpressed in many human cancers [9]. It regulates all hallmarks of cancer, including proliferation, mitosis, epithelial-mesenchymal transition, invasion, and metastasis [10]. Not surprisingly, previously published studies have shown the critical role of FOXM1 in breast tumorigenesis and resistance to chemotherapy. In a study by Yang et al. the stable overexpression of FOXM1 was found to promote metastasis of breast cancer cells in vivo through stimulating the transcription of SLUG (also known as SNAI2) which promotes the epithelial-mesenchymal transition in BC [11]. Xue and colleagues found that the activation of SMAD3/SMAD4 by FOXM1 promoted the TGF-β pathway activity and thus induced invasion and metastasis in BC [12]. The up-regulation of FOXM1 together with XIAP and Survivin antiapoptotic genes induces resistance in breast tumor cells to docetaxel, paclitaxel, and epirubicin [13]. In addition, FOXM1 is overexpressed in 85% of TNBCs [9] and is identified as the key transcriptional driver in the differentially expressed gene signature of TNBC [14]. FOXM1 promotes TNBC proliferation, invasion and progression by directly binding to and thus transcriptionally regulating expression of eEF2K [9]. FOXM1 also plays a role in autophagy by transcriptionally regulating *Beclin-1* and *LC3* genes in TNBCs [15]. Increased expression of the cAMP-response element-binding protein (CBP)/β-catenin/FOXM1 transcriptional complex in TNBC cells in vivo was associated with a high proportion of cancer stem cells, high rates of drug resistance and poor survival outcome [16]. In a recent study, Tan et al. constructed a gene regulation network in TNBCs and found that FOXM1 was in a key position in the network [17]. They further investigated the function of FOXM1 in the TNBC cell line MDA-MB-231 and found that inhibiting FOXM1 can significantly suppress MDA-MB-231 cell tumorigenesis in vivo using a mouse xenograft model [17]. Zhang et al. found that DEP (disheveled, EGL-10, pleckstrin) domain-containing (*DEPDC1*) was over-expressed in human TNBCs relative to their paired neighboring non-cancerous tissues using two public data sets from Gene Expression Omnibus (GEO) [18]. Stable DEPDC1 over-expression can facilitate cell proliferation and tumor growth via upregulating FOXM1 in MDA-MB-436 cells and BT549 cells [18]. Taken together, these results suggest that inhibition of FOXM1 function is a potential therapeutic strategy to treat TNBC.

The PPARA pathway is a predefined pathway extracted from the C2 category gene sets in the MSigDB database [8]. This pathway can induce tumor cell apoptosis and includes 57 gene members, such as the tumor suppressors *RB1* and *PIK3R1*, the proto-oncogenes *MYC* and *JUN*, as well as transcription factors *CITED2* and *PPARA*. The stimulation of the PPARA pathway increases the volume and number of peroxisomes which are responsible for, among other things, lipid metabolism and catabolism. Genes in this pathway are regulated by the PPARA transcription factor. Previous studies suggest that PPARA is a tumor suppressor in some cancers, including melanoma [19] and glioblastoma [20]. PPARA also appears to inhibit cell proliferation and tumorigenesis and induces degradation of the proto-oncogene Bcl2 which inhibits apoptosis in developing tumor cells [21]. Moreover, a group of co-expressed genes including *LPL*, *SORBS1*, *PPARG*, *PLIN*, *FABP4*, *AQP7*, *CD36*, and *ADIPOQ* that are involved in the PPARA signaling pathway may also inhibit the pathway and contribute to breast tumor progression [22]. Recently, Saleh et al. found that PD-L1 blockade by atezolizumab in the human TNBC cell line MDA-MB-231 downregulated tumor growth, metastasis, and hypoxia signaling pathways, including the PPARA/retinoid receptor a (RXRa) pathway [23]. To study the mechanisms by which adipose tissue in obesity promotes BC progression, Blucher et al. treated TNBC cells with adipose tissue conditioned media generated from the fatty tissue of obese female patients [24]. The adipose tissue treatment changed the expression profiles of TNBC cells resulting in altered expression of many genes regulated by PPAR nuclear receptors [24]. Thus, adipose tissue generated factors that altered PPAR-regulated gene expression and lipid metabolism, and further promoted TNBC progression. These results suggest that the PPAR pathway has potential targets that could be used to develop treatments for TNBCs [24].

The findings from our previous studies together with the published literature strongly support the roles of the FOXM1 and PPARA pathways in BC and especially TNBC tumorigenesis. Therefore, identifying compounds that can modulate these two pathways may provide novel therapeutic strategies for BC, and, in particular, TNBC. The purpose of this study was to investigate the suppression of the FOXM1 pathway and the stimulation of the PPARA pathway on BC cell lines with various compounds.

## Materials and methods

### Gene expression data and processing

**Fig. 1:**
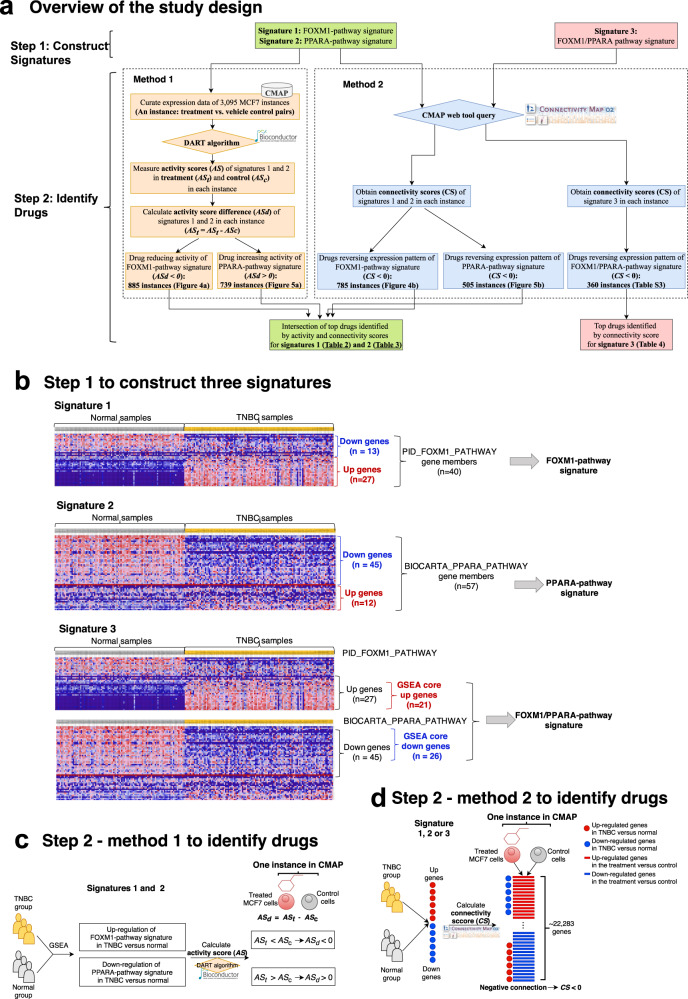
Study design. **a** The three major pipelines of this study are depicted. **b** Initially, three signatures were built: two signatures built with each of the FOXM1 and PPARA pathways alone and a third signature built with the two pathways together. **c** Next, the pathway activity scores of the FOXM1- and PPARA-pathway signatures based on genome-wide expression data with drug treatment were computed by the DART algorithm to identify drugs inducing the activity score changes of the two pathways. **d** In parallel, the connectivity scores for the FOXM1- and PPARA-pathway signatures were computed by the CMAP online tool to identify drugs reversing the expression pattern of the two pathway signatures. Finally, the intersection of the drugs identified from the two pipelines (**c**, **d**) were considered as drugs influencing the activity of the FOXM1 or PPARA pathways. In addition, connectivity scores for the FOXM1/PPARA-pathway signature were computed by CMAP online tool to identify drugs reversing expression pattern of the two pathways simultaneously.

The overall approach of this study is depicted in Fig. 1a. We collected gene expression data from three major resources. In order to assist in identifying novel compounds that might modulate the FOXM1 and PPARA pathways, the Connectivity Map (CMAP) database [25] was used in this study. CMAP has been widely used to identify novel therapeutic targets for a disease by establishing advantageous connections between the drug treatment and the patient response (phenotypic response) [26, 27]. Although it is not feasible to directly measure a pathway signaling activity, it can be approximated using gene expression. We used the CMAP build 02 database (http://www.broadinstitute.org/cmap), which includes 3095 drug-induced gene expression instances (treatment vs. vehicle control pairs) from MCF7 cells treated with 1294 bioactive small chemical molecules at varying concentrations. The raw CEL files were downloaded. Since the CMAP database is based on three different Affymetrix chip types (HG-U133A, HT_HG-U133A and U133AAofAv2), the microarray data were then grouped according to the platforms and the Robust Multichip Average (RMA) [28] method was used to normalize the drug-induced expression profiles from each chip type. Probe IDs were then mapped to gene symbols using the corresponding platform files. If a probe was mapped to multiple or zero genes, the data from this probe were discarded. If multiple probes were mapped to the same gene for a given expression profile, the maximum value from the probes was taken as the expression value for that gene. Finally, we kept only those genes present in all the three chip types and applied the *ComBat* function in the R package sva [29] to remove batch effects. Thus, the normalized and batch effect corrected drug-induced gene expression data from different platforms could be used for further study.

For the TCGA breast tumor data, we used the estimated baseline expression (i.e., without drug treatment) of genes computed by the RSEM method [30] provided by Firehose Broad GDAC (https://gdac.broadinstitute.org), multiplied by 10^6^ to obtain Transcripts Per Million (TPM) [30] and log2-transformed. The disease subtype for each sample was defined based on the tumor immunohistochemistry status with respect to ER, PR and HER2.

The GEO data set GSE48213 contains molecular profiles (pre-treatment measurements of mRNA expression, CNV, protein expression, promoter DNA methylation, and gene mutation) from a collection of 84 BC cell lines reported in Daemen et al.’s publication [31]. In this study, baseline expression data in Fragments Per Kilobase of transcript per Million mapped reads (FPKM) values that are available for 56 BC cell line samples were extracted from the data set GSE48213 [31], converted into TPM [32], and log-transformed log2(TPM + 1). Cell line, and subtype information was obtained from Daemen et al. [31]. Cell lines with expression data missing in more than 50% of all genes were excluded from this study.

### Pathway-based signature construction

Three gene signatures were constructed based on the GSEA results from the FOXM1 and PPARA pathways in our previous study [6] (Fig. 1b). A typical signature was represented by two subsets of genes (“up” tags and “down” tags). The FOXM1-pathway signature was built based on the expression pattern of the 40 gene members in the FOXM1 pathway between TNBC tumor samples and normal breast tissue samples observed in the GSEA results in our previous study [6] (Supplementary information: Table S1). We grouped those genes which are upregulated in TNBC relative to normal breast tissue into “up” while those genes which are downregulated in TNBC compared to normal breast tissue were grouped into “down” (Fig. 1b). Similarly, based on the expression pattern of the 57 gene members form the PPARA pathway between TNBC and normal breast tissue samples observed in the GSEA results in our previous study [6] (Supplementary information: Table S2), the PPARA-pathway signature was built. We grouped those genes which are upregulated in TNBC relative to normal breast tissue into “up” while those which are downregulated in TNBC compared to normal breast tissue were grouped into “down” (Fig. 1b).

Given that the FOXM1 pathway is the top upregulated pathway, while the PPARA pathway is the top downregulated pathway in TNBC versus normal breast tissue, the signatures from both pathways were simplified into a single FOXM1/PPARA-pathway signature by selecting genes that are most responsible for up-regulation of the FOXM1 pathway and down-regulation of the PPARA pathway (Fig. 1b). According to GSEA, for an enriched gene set, the core members contribute most to the gene set enrichment [33]. Thus, the core members of the FOXM1 pathway which accounted for the up-regulation of the FOXM1 pathway in TNBC versus normal (Supplementary information: Table S1) were selected and tagged as “up”. The core members of the PPARA pathway which accounted for the down-regulation of the PPARA pathway in TNBC versus normal (Supplementary information: Table S2) were selected and tagged as “down”. Thus, the FOXM1/PPARA pathway signature represents the core gene members from the FOXM1 and the PPARA pathways.

### Pathway activity score calculation

To calculate the activity score of the FOXM1- and PPARA-pathway signatures, the *DoDART* function from the De-noising Algorithm based on Relevance network Topology (DART) [34] R package was used. By inputting the expression profiles of given samples and a predefined signature, the *DoDART* function performed the following steps to calculate pathway activity scores using the DART algorithm: 1) constructing the relevance network where nodes are genes in the signature and edges represent the correlation among genes based on the expression profiles. The significance of the correlation between a gene pairs is defined using the default FDR value of 1.0 ×10^−6^, which is stringent and represents a conservative Bonferroni threshold that assumes that a typical signature consists of on the order of 100 genes thereby necessitating an estimated 10,000 pairwise gene correlations; 2) evaluating the consistency of the gene-gene correlations (i.e., edges) in the relevance network with the prior gene-gene correlations contained in the given signature; 3) filtering out edges that are inconsistent with the prior information contained in the given signature from the relevance network; and 4) estimating an activity score of the given signature for each individual sample using its expression profile. A high positive pathway activity score indicates the stimulation of the pathway activity in a given sample while high negative score indicates the repression of the pathway activity.

Considering the difference between cancer tissue and cell lines in terms of transcriptional profiles, we asked whether the activity scores of the FOXM1- and PPARA-pathway signatures across different BC subtypes showed a similar pattern between the BC tissue and cell lines. We computed the activity scores of the two signatures for the BC cell line samples from the GSE48213 gene expression data set and also for BC tissue samples from the TCGA BC gene expression data set. The activity scores of the two pathways in the BC tissue and cell lines were normalized into the range of 0 to 1 for easy comparison. For each signature, we performed the ANOVA test to check the difference of activity score in three BC cell line subtypes (luminal, HER2 and TNBC) in GSE48213. To compare the means of the signature activity score between any two BC cell line subtypes in GSE48213, we performed pairwise t-tests between every two subtypes followed by Benjamini–Hochberg correction for multiple testing. For each signature, we also performed the ANOVA test to check the difference of the signature activity score in five BC tissue sample groups (luminal A, luminal B, HER2-enriched, TNBC, and normal breast tissue) in TCGA. We performed pairwise t-tests between BC tissue pairs followed by Benjamini–Hochberg correction for multiple testing to compare the means of the pathway activity scores between BC tissue sample groups in TCGA.

To assess the effect of a treatment on the activity of the FOXM1 or PPARA pathways, we applied the *DoDART* function to the CMAP perturbational expression profiles and calculated the FOXM1- and PPARA-pathway signature activity scores for each CMAP instance (i.e., the treatment and the paired vehicle control) (Fig. 1c). In the case of multiple controls per treatment, we removed the control with the highest and lowest activity scores as outliers, and then used the mean of the rest as the control. Some compounds with the same dose were exposed to the MCF7 cells for multiple times. In this case, we took the average of the activity scores of the FOXM1 and PPARA pathways. In the end, activity scores of the two pathways for 1390 MCF7 gene expression instances (i.e., treatment vs. vehicle control pairs) were obtained.

### Identification of compounds modulating pathway activity scores

We evaluated compounds, many of which are drug treatments, affecting pathway activity in the context of the change of pathway activity score between treated and control cell lines in CMAP (Fig. 1c). For each of the two pathways in a given instance, we defined the activity score difference (*AS*_*d*_) as the activity score in the treatment (*AS*_*t*_) minus the activity score in the control (*AS*_*c*_) (Eq. (1)).

1 $$AS_d = AS_t - AS_c$$

The magnitude of *AS*_*d*_ is the degree of change in pathway activity score caused by the corresponding treatment, while the sign of *AS*_*d*_ is the direction of that change. Therefore, a positive sign indicates the treatment increases the pathway activity while a negative sign decreases the pathway activity. We ordered the instances according to increasing difference score for the FOXM1 pathway but decreasing difference score for the PPARA pathway. Therefore, the top-ranked instances for the FOXM1 pathway are those which decrease the pathway activity while for PPARA pathway are those which increase the pathway activity. These top-ranked instances were further investigated.

### Connectivity Map query of compounds modulating pathway expression patterns

In comparison to drug treatments affecting pathway activity in the context of the change of pathway activity score between treated and control cell lines in CMAP, we also evaluated drug treatments in the context of modulating (mimicking or reversing) pathway expression patterns using the CMAP online tool (Fig. 1d). The three signatures (the FOXM1-pathway signature, the PPARA-pathway signature, and the FOXM1/PPARA-pathway signature) were used as the query signatures to perform the CMAP analysis through the CMAP build 02 web interface. To query the CMAP online tool with a given signature, we first changed the gene list from gene symbols to Affymetrix probe IDs, which are required as input into the CMAP. This probe list was collated into tag sets of “up” or “down” genes and queried against the CMAP database to generate hits. The similarity between the gene expression profile of the query signature and that of a CMAP instance was measured by the connectivity score, which ranged from −1 to 1. When a query signature receives a high positive score for a treatment, it means with this treatment the upregulated (i.e., “up”) genes in the signature are also upregulated while the downregulated (i.e., “down”) genes in the signature are also downregulated. Thus, a high positive connectivity score indicates that the corresponding compounds induced the same changes in expression of the query signature as those caused by BC. When a query signature receives a high negative score for a treatment, it means that the upregulated genes in the query signature are downregulated by the treatment while the downregulated genes in the signature are upregulated by the treatment (Fig. 1d). Therefore, a high negative connectivity score indicates that the corresponding compounds reversed the expression of the query signature. In the current study, for a query signature, we expected the upregulated genes to be downregulated while the downregulated genes to be upregulated after a particular treatment. So those treatments returning a large negative connectivity score for the three query signatures are treatments of interest. In other words, we expected to identify the compounds which could reverse the expression patterns of the three signatures.

## Results

### Three pathway-based signatures

Gene lists for the three signatures are provided in Table 1. According to the GSEA results [6] (Supplementary information: Table S1), of the 40 genes in the FOXM1 pathway, 27 were upregulated and 13 downregulated in TNBC compared to normal breast tissue. Thus, the 40-gene FOXM1 pathway signature was represented by two subsets of genes (“up” tags and “down” tags). In the same way, the 57 genes in the PPARA pathway were divided into two parts, 12 up, and 45 downregulated genes (Supplementary information: Table S2). It is noteworthy that the majority of the FOXM1 pathway genes are upregulated while the majority of the PPARA pathway genes are downregulated in TNBC, which is in accordance with the up-regulation of the FOXM1 pathway and the down-regulation of the PPARA pathway in TNBC identified by the GSEA analysis in our previous study [6]. In this way, we built the FOXM1-pathway signature and the PPARA-pathway signature (Fig. 1b).

**Table 1:** The three signatures established by using FOXM1 and PPARA pathways.

Signature	Gene member	Gene number	Tag
FOXM1-pathway signature	*AURKB, BIRC5, BRCA2, CCNA2, CCNB1, CCNB2, CCND1, CCNE1, CDC25B, CDK1, CDK2, CDK4, CDKN2A, CENPA, CENPB, CENPF, CHEK2, CKS1B, ESR1, FOXM1, GSK3A, HIST1H2BA, NEK2, ONECUT1, PLK1, SKP2, XRCC1*	27	up
	*CREBBP, EP300, ETV5, FOS, GAS1, LAMA4, MAP2K1, MMP2, MYC, NFATC3 RB1, SP1, TGFA*	13	down
PPARA-pathway signature	*APOA1, APOA2, DUT, HSP90AA1, HSPA1A, INS, MED1, MRPL11, NCOR2, NR0B2, RELA, SRA1*	12	up
	*ACOX1, CD36, CITED2, CPT1B, CREBBP, DUSP1, EHHADH, EP300, FABP1, FAT1, HSD17B4, JUN, LPL, MAPK1, MAPK3, ME1, MYC, NCOA1, NCOR1, NFKBIA, NOS2, NR1H3, NR2F1, NRIP1, PDGFA, PIK3CA, PIK3CG, PIK3R1, PPARA, PPARGC1A, PRKACB, PRKACG, PRKAR1A, PRKAR1B, PRKAR2A, PRKAR2B, PRKCA, PRKCB, PTGS2, RB1, RXRA, SP1, STAT5A, STAT5B, TNF*	45	down
FOXM1/PPARA-pathway signature	*AURKB, BIRC5, BRCA2, CCNA2, CCNB1, CCNB2, CCNE1, CDC25B, CDK1, CDK2 CDK4, CDKN2A, CENPA, CENPF, CHEK2, CKS1B, FOXM1, GSK3A, NEK2, PLK1, SKP2*	21	up
	*STAT5B, HSD17B4, PIK3R1, DUSP1, CD36, CITED2, STAT5A, SP1, PRKAR1A, NCOA1, JUN, MAPK3, LPL, PRKAR2B, NCOR1, RB1, RXRA, NR2F1, EHHADH, ACOX1, PRKACB, NRIP1, PDGFA, NR1H3, CREBBP, PRKAR2A*	26	down

Given the trend of up-regulation of the FOXM1 pathway and down-regulation of the PPARA pathway in TNBC, the signatures from both pathways were simplified into a single FOXM1/PPARA pathway signature by identifying genes that are most responsible for FOXM1 pathway up-regulation and PPARA pathway down-regulation. Among the 27 upregulated genes from the FOXM1 pathway, 21 were core members that contribute most to the pathway up-regulation in the GSEA results in our previous study [6] (Supplementary information: Table S1). Among the 45 downregulated genes from the PPARA pathway, 26 were core members that contribute most to the pathway down-regulation in the GSEA results in our previous study [6] (Supplementary information: Table S2). We used the 21 core gene members from FOXM1 pathway as the “up” genes and the 26 core gene members from the PPARA pathway as the “down” genes to build the FOXM1/PPARA-pathway signature (Fig. 1b).

### FOXM1 pathway activity in breast cancer tissue and cell line samples

**Fig. 2:**
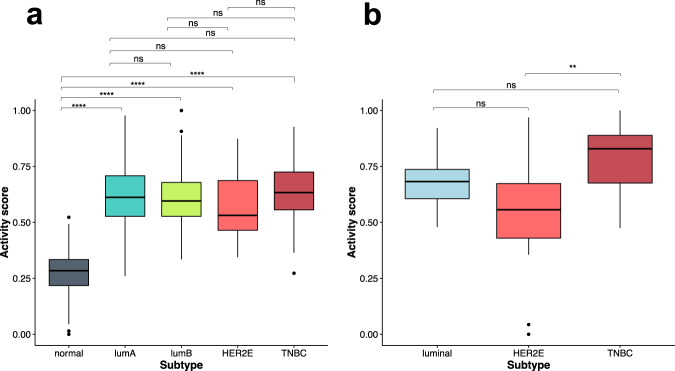
The FOXM1 pathway activity score in breast cancer. **a** The FOXM1 pathway activity score in TCGA BC tissue samples. **b** The FOXM1 pathway activity score in GSE48213 BC cell line samples. The pairwise comparison between groups using t-tests followed by Benjamini–Hochberg correction. The significance level of adjusted *p*-values is as follows: ns, *p* > 0.05; *p ≤ 0.05; **p ≤ 0.01; ***p ≤ 0.001; ****p ≤ 0.0001.

We calculated the activity score of the FOXM1 pathway for TCGA breast tumors [35], including 424 luminal A, 121 luminal B, 37 HER2-enriched, 112 TNBC, and 112 normal breast tissue samples. There are significant differences among the five groups regarding the FOXM1 pathway activity score (ANOVA, *p*-value < 2.2 × 10^−16^) (Fig. 2a). We found that the averaged activity score is significantly lower in the normal breast tissue group than each of the four TCGA BC groups (luminal A, luminal B, HER2-enriched, and TNBC) (Fig. 2a). Among the four TCGA BC subtypes, the TNBC group appears to show a higher FOXM1 activity score than HER2-enriched but the results are not statistically significant (Fig. 2a). We note that the GSEA results in our previous study [6] showed the FOXM1 pathway was the most significantly upregulated pathway in TNBC.

Although there are well documented differences between BC cell lines and BC tissue samples, we expected to see at least some similarity between their pathway activity scores given that BC cell lines are ultimately derived from BC tissue samples. With the gene expression data and subtype annotation from the publicly available GEO data set GSE48213 [31], we obtained data for 14 luminal, 21 HER2-enriched and 10 TNBC cell lines. The GSE48213 data set included two matched normal-like BC lines, on which RNA-Seq was not performed and thus were excluded. We calculated the activity scores of FOXM1 pathway for the GSE48213 BC cell lines. The FOXM1 pathway activity scores showed a significant difference among the three GSE48213 BC cell line subtypes (ANOVA, *p*-value = 0.003) (Fig. 2b). Similar to the results in the four TCGA BC subtypes (Fig. 2a), the TNBC cell line group exhibits a higher FOXM1 activity score than the luminal and HER2-enriched cell line groups (Fig. 2b). There is a significant difference between the FOXM1 pathway activity scores for the TNBC and HER2-enriched subtypes but no significant difference between the TNBC and luminal subtypes. The similarity of the FOXM1 pathway activity score in TCGA breast tumor tissue samples and GSE48213 BC cell lines suggests that the compounds that can modulate the FOXM1 pathway in BC cell lines could also potentially modulate the pathway in breast tumor tissue samples.

### PPARA pathway activity in breast cancer tissue and cell line samples

**Fig. 3:**
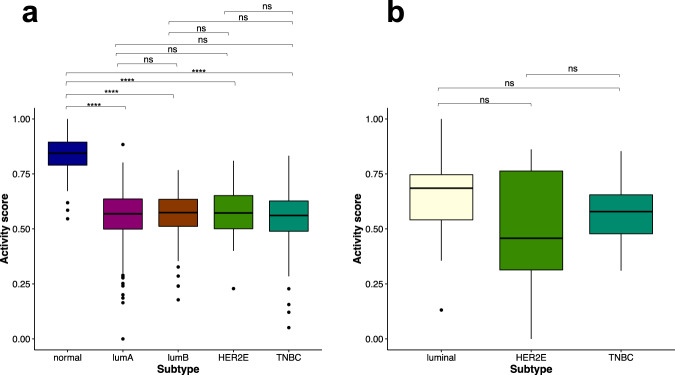
The PPARA pathway activity score in breast cancer. **a** The PPARA pathway activity scores from TCGA BC tissue samples. **b** The PPARA pathway activity score in GSE48213 BC cell lines. Pairwise comparisons between groups were performed with t-tests followed by Benjamini–Hochberg corrections. The significance level of adjusted *p*-values is as follows: ns, *p* > 0.05; *p ≤ 0.05; **p ≤ 0.01; ***p ≤ 0.001; ****p ≤ 0.0001.

The activity scores of the PPARA pathway were calculated for breast tumor samples from TCGA and BC cell lines from GSE48213. The PPARA pathway activity score is significantly different among the five TCGA BC tissue groups (ANOVA, *p*-value < 2.2 × 10^−16^) (Fig. 3a). The normal tissue group from TCGA shows a significantly higher PPARA pathway activity score than each of the four breast cancer tissue groups (Fig. 3a). However, no significant difference in the PPARA pathway activity score was observed among the four TCGA BC subtypes. Similarly, the activity of the PPARA pathway does not show significant differences among the three GSE48213 BC cell line subtypes (ANOVA, *p*-value = 0.15) (Fig. 3b), similar to the results observed in TCGA breast tumor tissue samples. The similarity of the PPARA pathway activity score in TCGA breast tumor tissue samples and GSE48213 BC cell lines suggests that the compounds that can modulate the PPARA pathway in BC cell lines could also potentially modulate the pathway in breast tumor tissue samples.

### Identification of compounds modulating the FOXM1 pathway activity in breast cancers

**Fig. 4:**
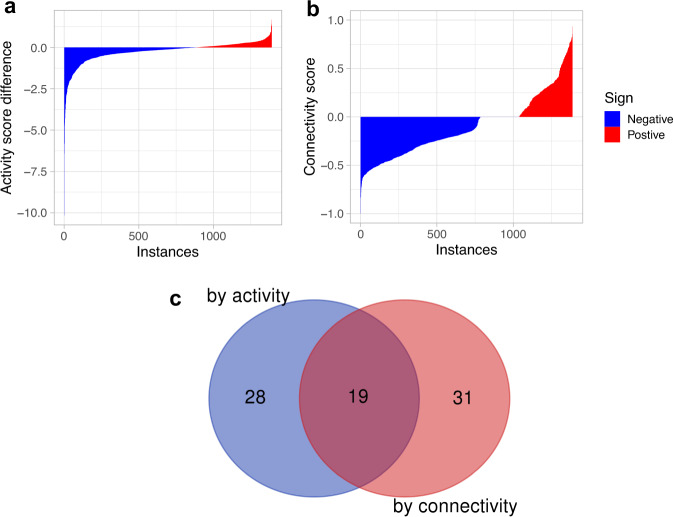
Affects of compounds (i.e., instances) on the FOXM1 pathway activity and expression pattern. **a** The FOXM1 pathway activity score differences derived from the 1390 CMAP gene expression instances. The y-axis shows the difference between the activity score in the treatment and the paired control for each instance. The x-axis shows the instances ordered by increasing corresponding activity score difference. **b** The connectivity score of the 1390 CMAP gene expression instances where the y-axis shows the connectivity score and the x-axis shows the instances ordered by increasing corresponding connectivity score. **c** A Venn diagram showing the intersection between the top 50 drugs identified by the activity score difference and the activity score.

To identify drugs that can modulate the FOXM1 pathway activity in BC we used the CMAP database. CMAP contains 1390 drug-induced gene expression instances from treatment vs. vehicle control pairs in MCF7 cells treated with 1294 approved drugs and experimental compounds. We evaluated the effect of these drugs on the FOXM1 pathway activity score (Fig. 1c). Since the FOXM1 pathway has a high positive activity score (i.e., activated) in BC while a high negative activity score (i.e., inactivated) in normal breast tissue, an effective drug treatment should reduce the FOXM1 pathway activity score (i.e., suppressing the pathway activity) and thus have a larger negative activity score difference (*AS*_*d*_) between treatment and control groups. Of the 1390 perturbational gene expression instances, 885 instances show a negative difference score for the FOXM1 pathway (i.e., the activity score in the treatment is less than the paired control) (blue bars in Fig. 4a), suggesting that the corresponding treatment suppresses the FOXM1 pathway activity. For comparison, we also evaluated the ability of the drug treatments to reverse the pattern of gene expression in the FOXM1 pathway (Fig. 1d). In parallel, out of the 1390 expression instances, 785 generated a negative connectivity score in the CMAP query results for the FOXM1 pathway (blue bars in Fig. 4b), suggesting that the corresponding treatment could reverse the pattern of gene expression in the FOXM1 pathway. Ordering the instances according to increasing activity difference and connectivity scores, respectively, we examined the top 50 instances in detail. After extracting the top 50 instances from each case, we found that 19 drugs decreased the FOXM1 pathway activity scores and reversed the pattern of gene expression in the FOXM1 pathway simultaneously (Fig. 4c, Table 2).

**Table 2:** The intersection of top 50 drugs by activity (Method 1) and connectivity (Method 2) scores on FOXM1 pathway.

Drug name	Dose (µM)	*N*^a^	Rank by		Activity score (Method 1)			Connectivity score (Method 2)
			Activity score difference (*AS*_*d*_)^b^	Connectivity score	Treatment (*AS*_*t*_)	Control (ASc)	Difference (ASd)	
5109870	25	1	1	10	−8.57	1.59	−10.16	−0.66
MG-132	21	1	2	1	−4.45	1.97	−6.42	−1
MG-262	0.1	1	4	3	−2.95	1.79	−4.74	−0.83
Celastrol	2.5	1	5	7	−2.99	1.59	−4.58	−0.72
Resveratrol	50	2	6	8	0.92	5.2	−4.28	−0.7
Ciclopirox	15	2	7	37	−2.45	1.53	−3.98	−0.59
Pyrvinium	3.4	2	11	2	−1.85	1.56	−3.41	−0.84
Emetine	7.2	2	13	14	−1.37	1.71	−3.08	−0.63
15-delta prostaglandin J2	10	8	14	11	−1.18	1.73	−2.91	−0.66
Cephaeline	6	3	15	32	−0.95	1.96	−2.91	−0.6
Puromycin	7.4	2	16	17	−0.94	1.89	−2.84	−0.62
Parthenolide	16.2	2	23	18	−1.43	1.03	−2.46	−0.62
Azacitidine	16.4	2	24	49	−0.87	1.53	−2.39	−0.57
Cycloheximide	14.2	2	28	45	−0.3	2	−2.29	−0.57
Astemizole	8.8	2	29	27	0.04	2.3	−2.26	−0.6
5224221	12	2	36	25	−1.42	0.61	−2.03	−0.6
Scriptaid	10	1	42	20	1.32	3.29	−1.97	−0.62
Ouabain	5.4	2	43	30	−0.34	1.63	−1.97	−0.6
Bepridil	10	2	49	13	0.12	2	−1.88	−0.63

^a^The number of experiments.

^b^Activity difference (*ASd*) = activity score in treatment (*ASt*) − activity score in control (*ASc*).

### Identification of compounds modulating the PPARA pathway activity in breast cancers

**Fig. 5:**
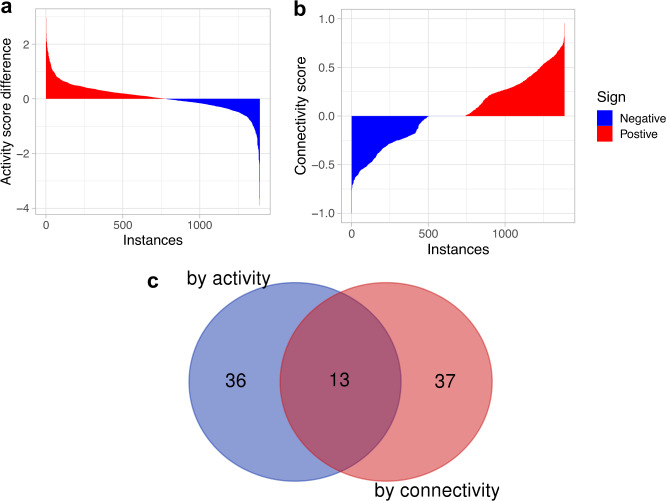
Affects of compounds (i.e., instances) on the PPARA pathway activity and expression pattern. **a** The PPARA pathway activity score difference of the 1390 CMAP gene expressoin instances. The y-axis shows the difference between the activity score in the treatment and the paired control for each instance. The x-axis shows the instances ordered by decreasing corresponding activity score difference. **b** The connectivity score of the 1390 CMAP gene expression instances. The y-axis shows the connectivity score and the x-axis shows the instances ordered by increasing corresponding connectivity score. **c** A Venn diagram showing the intersection between the top 50 drugs identified by the activity score difference and the activity score.

We evaluated the effect of drug treatments on PPARA pathway activity scores (Fig. 1c). The PPARA pathway shows a high negative activity score (i.e., inactivated) in BC tumors and a high positive activity score (i.e., activated) in normal breast tissue. Thus, an effective drug treatment would be able to increase the PPARA pathway activity score (i.e., stimulating the pathway activity) and thus has a larger positive activity score difference (*AS*_*d*_) between the treatment and the paired control groups. Of the 1390 drug-induced gene expression instances, 739 show a positive difference score for the PPARA pathway (i.e., the activity score in the treatment is larger than the paired control) (red bars in Fig. 5a), suggesting that the corresponding treatment increases the PPARA pathway activity. For comparison, we also evaluated the ability of the drug treatments to reverse the pattern of gene expression in the PPARA pathway (Fig. 1d). The CMAP query returned 505 instances with a negative connectivity score for the PPARA pathway out of the 1390 instances (blue bars in Fig. 5b), suggesting that the corresponding treatments could reverse pattern of gene expression in the PPARA pathway. Ordering the instances according to increasing activity difference and connectivity scores, respectively, and after extracting the top 50 instances from each case, we found that 13 drugs increased the PPARA pathway activity scores and reversed the pattern of gene expression in the PPARA pathway simultaneously (Fig. 5c, Table 3).

**Table 3:** The intersection of top 50 drugs by activity (Method 1) and connectivity (Method 2) scores on PPARA pathway.

Drug name	Dose (µM)	*N*^a^	Rank by		Activity score (Method 1)			Connectivity score (Method 2)
			Activity score difference (*ASd*)^b^	Connectivity score	Treatment (*AS*_*t*_)	Control (*ASc)*	Difference (*ASd*)	
Anisomycin	15	2	1	15	2.71	−0.26	2.96	−0.66
Cephaeline	6	3	2	48	3.01	0.61	2.4	−0.58
Pararosaniline	10	1	3	4	2.52	0.3	2.22	−0.73
Cycloheximide	14.2	2	6	39	2.05	−0.02	2.08	−0.61
Monensin	10.9	1	17	21	0.34	−1.23	1.57	−0.64
Wortmannin	1	2	18	44	2.75	1.18	1.56	−0.6
Raloxifene	0.1	1	24	47	0.72	−0.66	1.38	−0.58
Prednisolone	1	1	25	8	0.65	−0.73	1.38	−0.71
Valinomycin	0.1	2	34	29	1.11	−0.09	1.2	−0.62
Oligomycin	1	1	37	31	0.51	−0.63	1.15	−0.62
Mepacrine	7.8	1	44	14	0.93	−0.1	1.03	−0.66
5186324	2	1	47	20	1.31	0.3	1.01	−0.65
5162773	7	1	48	30	1.31	0.3	1.01	−0.62

^a^The number of experiments.

^b^Activity difference = activity score in treatment (ASt) − activity score in control (ASc).

### Identification of compounds modulating the FOXM1 and PPARA pathways concurrently in breast cancers

Using the FOXM1/PPARA pathway signature built using both FOXM1 and PPARA pathways to query the CMAP database, we identified 360 compounds (Supplementary information: Table S3) that can reverse the pattern of gene expression in the FOXM1 and PPARA pathways in BC. The top 10 such drugs, with the smallest connectivity scores, are listed in Table 4. To have a further look at the effect of these CMAP gene expression instances on the FOXM1 and PPARA pathways, we also include the up-scores representing the absolute enrichment of the up-genes (i.e., the “core” genes which are upregulated in BC and from the FOXM1 pathway) in a given instance and the down-scores representing the absolute enrichment of the down-genes (i.e., the “core” genes which are downregulated in the PPARA pathway in BC) in a given instance. Both the two types of scores can adopt values between +1 and −1. A high positive up (or down)-score indicates that the corresponding drug induced the expression of the up (or down)-genes. Whereas a high negative up (or down)-score indicates that the corresponding drug repressed the expression of the up (or down)-genes. We therefore expected that drugs that might be effective in BC would repress the expression of the up-genes while inducing the expression of the down-genes. Thus, the most effective drugs were expected to have a high negative up-score and a high positive down-score. Among the top 10 drugs, MG-262, MG-132, celastrol, ciclopirox, and puromycin, which are also among the top drugs from the FOXM1 pathway, showed a negative correlation with the FOXM1/PPARA pathway signature, indicating their ability to reverse the pattern of gene expression in the FOXM1 and PPARA pathways in BC. Examination of the up- and down-scores shows that these compounds produce a higher magnitude repression of the FOXM1 pathway than their stimulation of the PPARA pathway (Table 4).

**Table 4:** The top 10 drugs identified by Method 2 for downregulating the FOXM1 and upregulating the PPARA pathways simultaneously.

Rank	Drug name	Dose (µM)	*N*^a^	Up score	Down score	Connectivity score (Method 2)
1	MG-262	0.1	1	−0.55	0.19	−0.91
2	Clotrimazole	50	1	−0.32	0.34	−0.82
3	MG-132	21	1	−0.52	0.14	−0.81
4	Hycanthone	11	2	−0.44	0.15	−0.73
5	Celastrol	3	1	−0.43	0.15	−0.72
6	Ciclopirox	15	2	−0.46	0.11	−0.71
7	Withaferin A	1	2	−0.39	0.18	−0.7
8	Cephaeline	6	3	−0.35	0.19	−0.67
9	Pararosaniline	10	1	−0.31	0.23	−0.67
10	Puromycin	7	2	−0.34	0.19	−0.66

^a^The number of the MCF7 cell samples treated by the drug at the same dose.

## Discussion

CMAP analysis has been widely used to identify novel therapeutic treatments and it can be performed by querying a pathway signature against the CMAP database using the gene set enrichment analysis algorithm described by Lamb et al. [25]. The generated connectivity score is an indication of the ability of a given drug to reverse or induce the expression pattern of the queried pathway. This method considers all gene members in a pathway of equal relevance and treats pathways as unstructured lists of genes. The *DART* algorithm evaluates the relevance of the prior information of genes in a given pathway and signature and then estimates pathway activity. In this study, we identified the drug candidates for their ability to modulate the FOXM1 and PPARA pathways using both the CMAP query and the DART algorithm. The intersection of drugs identified from each method increased our confidence in their identification as candidates.

Among the 19 drugs (Table 2) identified by both the CMAP query and the DART algorithm as repressing the FOXM1 pathway, some have been noted in other studies for their potential to treat cancer. For example, the ChemBridge compound 5109870 which had the lowest activity score difference (−10.16) and was ranked tenth by the connectivity has been found previously to induce HIF-1α-responsive genes, chelate iron, and block progression of BC in two distinct mouse models [36]. MG-132 and MG-262 are proteasome inhibitors which have been shown in vitro and in vivo to have anticancer properties on their own, or synergistically with other compounds [37]. In one study, the combination of natural compounds such as gambogic acid with MG-132 or MG-262 showed inhibitory effects on growth of malignant cells and tumors in allograft animal models apparently with no observed systemic toxicity [38]. Celastrol is a compound derived from *Tripterygium wilfordii* root and was reported to inhibit breast cancer cell invasion by reducing NF-ĸB-mediated matrix metalloproteinase-9 expression [39] and also showed anticancer effects on human TNBC, potentially by affecting oxidative stress, apoptosis and the PI3K/Akt pathways [40]. Several studies have suggested that resveratrol may have anti-tumor affects through a variety of mechanisms including but not limited to inhibition and/or activation of histone deacetylases [41] and suppression of the PI3K/Akt signaling pathway [41]. In particular, resveratrol inhibits migration of MDA-MB-435 BC cells via suppression of the PI3K/Akt signaling pathway [41]. In BC, it is reported that the proliferating cell percentage was reduced with resveratrol. In addition, higher doses of resveratrol delayed tumor formation and multiplicity, while the lower dose did not significantly alter these parameters when compared to a control. The work of Chatterjee et al. reported that resveratrol decreased the expression of TGFβ1 and NF-κB and the expression and activity of 5-LOX, as well as cell proliferation, while increasing the faction of cells undergoing apoptosis [42]. Resveratrol also decreased the appearance of single-strand DNA, suggesting that there was less DNA damage [42]. Despite these interesting findings, it should be noted that multiple studies on resveratrol have demonstrated its inconsistent effects on cancer in animals and humans including negative, positive and no effect on cancer outcomes [41, 43]. Ciclopirox, which is currently used as a topical antifungal, may also inhibit cell proliferation, inducing cell death, as well as inhibiting angiogenesis and lymph angiogenesis all of which suggest a potential role as an anti-cancer drug. Zhou et al. showed that ciclopirox inhibits human breast cancer MDA-MB-231 growth in xenografts [44]. Emetine, which is currently used as an antiemetic and for the treatment of amebiasis, has also demonstrated anticancer properties, by inhibiting both ribosomal and mitochondrial protein synthesis and interfering with the synthesis and activities of DNA and RNA [45].

We found 13 drugs (Table 3) that could increase the PPARA pathway activity scores by the DART algorithm and reverse the PPARA pathway expression pattern by the CMAP query. Anisomycin is an antibiotic which is active against protozoa and yeast by inhibiting DNA and protein synthesis [46]. Recently, anisomycin has been found to be active against certain types of cancer, such as ovarian cancer, colon cancer and renal carcinoma [46]. It is also an agonist of p38-mitogen activated-protein kinase and c-Jun N-terminal kinase [46]. Anisomycin has been shown to sensitize glucocorticoid-resistant leukemia cells to dexamethasone-induced apoptosis through p38-MAPK/JNK [46]. Prednisolone is a glucocorticoid anti-inflammatory similar to dexamethasone. It has been used in combination with other anticancer drugs to treat some kinds of leukemia and lymphoma and to reduce the incidence of anemia and thrombocytopenia caused by cancer treatments. Interestingly, raloxifene is a SERM that produces anti-estrogenic effects in breast and uterine tissues and is used to decrease the risk of BC in post-menopausal women who are at high risk for invasive BC [47, 48].

Among the top 10 drugs (Table 4) that induce the PPARA pathway and repress the FOXM1 pathway concurrently, clotrimazole shows a connectivity score of −0.82, together with a similar up score and down score, suggesting its capacity to suppress the FOXM1 pathway while inducing the PPARA pathway. Clotrimazole is a widely used topical antifungal that has been found to preferentially inhibit human BC cell proliferation, viability and glycolysis [49]. Hycanthone and withaferin A were found to reverse the pattern of gene expression in the FOXM1 and PPARA pathways like clotrimazole but unlike clotrimazole, these two compounds have a stronger effect on the FOXM1 pathway than on the PPARA pathway. Hycanthone is an antischistosomal that is a thioxanthone derivative, and was found to exhibit, antitumor, and anti-metastatic activities against BC, however, it also causes life threatening liver toxicity [50]. Two compounds closely related to hycanthone, SR271425 and SR233377 are in Phase I and II clinical trials for solid tumors but appear to have less liver toxicity. Withaferin A is a steroidal lactone and negatively regulates breast cancer growth [51]. Pararosaniline is a dye that was among the top drugs for the PPARA pathway and could also reverse the pattern of gene expression in the FOXM1 and PPARA pathways. Cephaelin, with a connectivity score of −0.67, was among the top drugs for both the FOXM1 and PPARA pathways (Tables 2 and 3) and therefore reversed the pattern of gene expression in the FOXM1 and PPRA pathways (Table 4). MG-262, MG-132, celastrol, ciclopirox, and puromycin, which are also among the top drugs for the FOXM1 pathway, showed a negative correlation with the FOXM1/PPARA pathway signature, indicating they could reduce the FOXM1 pathway activity and increase the PPARA pathway activity. Examination of the up- and down-scores show that these compounds produce a greater repression of the FOXM1 pathway than their stimulation of the PPARA pathway. A similar trend was observed for all compounds in Table 4.

Cancer cell lines have been used extensively to screen anti-cancer drug candidates. However, the capacity of candidate drugs to repress tumor cells in vitro is not quantified as often. In this study, we investigated the activity pattern of the FOXM1 and PPARA pathways in both TCGA breast tumor patient and breast cancer cell line data (Figs. 2 and 3). The FOXM1 pathway has a higher activity score in the four BC subtypes than that in the normal tissue samples, with the highest in TNBC. The PPARA pathway has a higher activity score in the normal breast tissue samples than that in the four BC subtypes. No significant difference was found among the four BC groups for their PPARA pathway activity. With the BC cell lines, we were surprised to see that the distribution of the FOXM1 and PPARA pathway activity scores in breast tumor cell lines is similar to that in TCGA breast tumor patients. The findings suggested that drugs affecting the two pathways in BC cell lines could also potentially affect the two pathways in the BC tumors.

It is difficult to draw any conclusions about the clinical relevance of doses used in Tables 2 through 4, since the data from these tables derive from experiments performed with cultured MCF7 cells and not humans. Accordingly, the environment of such experiments is substantially different from the human systemic circulation, and in any case, it is unlikely that the plasma concentration and the tumor concentration of any drug are the same. Moreover, many of the drugs in Tables 2 through 4 are experimental and being as they are untested in humans, we lack any relevant human plasma concentration data. The antifungals clotrimazole and ciclopirox (Tables 2–4) are only administered topically in humans so we would not expect any significant systemic absorption and again, lack relevant clinical concentration data. However, some of the drugs identified in Tables 2 through 4 have such clinical data. For example, when taken orally at normal doses, the typical plasma concentration of raloxifene is 0.0028 μM. The dose used in Table 3 is 0.1 μM which is >30-fold higher and may reflect the need for a higher doses to induce cell death in MCF7 cells. The maximum plasma concentrations of prednisolone (Table 3) after oral administration varies widely based on dose but is typically between 0.3 and 3.7 μM and the 1 μM dose used in Table 3 is within this range.

The limitation of this study was that only the MCF7 cell line was used to investigate the drug candidates for the two pathways. MCF7 is known to be luminal A breast tumor cell line and therefore cannot fully represent each sub-type of breast cancer, which is a complex and heterogeneous disease. Therefore, the drugs identified for the FOXM1 and PPARA pathways in MCF7 cells may not have the same effects in the luminal B, HER2-enriched, and TNBC subtypes. Ideally, we would have been able to identify the drugs for the two pathways in cell lines representing each BC subtype, but we did not have this data. Nevertheless, it is interesting to note that MCF7 cells grown continuously in the absence of estrogen or the presence of SERMs or estrogen antagonists begin to from sub-populations that are triple negative (TN) [52]. Furthermore, evidence suggests that this is a result of overgrowth of minor TN sub-populations within MCF7 cells rather than differentiation of the parental MCF7 cell line [53]. Such data suggest that MCF7 cells used to generate the data in this study may have had sub-populations with a TN phenotype.

## Conclusions

Up-regulation of the FOXM1 pathway and down-regulation of the PPARA pathway were found in BCs and in particular TNBCs. Therefore, the current study was aimed to identify compounds effective at repressing the FOXM1 pathway activity as well as those inducing the PPARA pathway activity in BC. The former included 5109870, MG-132, MG-262, celastrol, resveratrol, ciclopirox and cephaeline while the latter included anisomycin, cephaeline, pararosaniline cicloheximide and monensin. In addition, compounds decreasing the FOXM1 pathway activity while increasing the PPARA pathway activity concurrently were identified, including MG-262, MG-132, celastrol, ciclopirox and puromycin.

## Supplementary information

Table S1

Table S2

Table S3

## Data Availability

The datasets analyzed during the current study are publicly available. The perturbational gene expression data for treatment-control pairs can be downloaded from the CMAP build 02 database (http://www.broadinstitute.org/cmap). The TCGA breast tumor patient gene expression data are available in the Firehose Broad GDAC (https://gdac.broadinstitute.org). The breast cancer cell line gene expression dataset GSE48213 is available in the GEO repository (https://www.ncbi.nlm.nih.gov/geo/).

## Code availability

The code used for carrying out the analyses in the study can be requested from the authors.
